# Early-Onset Normal-Tension Glaucoma in Patients Under 40 Years of Age: Risk Factors for Progression

**DOI:** 10.7759/cureus.106455

**Published:** 2026-04-05

**Authors:** Aparna Rao, Sirisha Senthil

**Affiliations:** 1 Glaucoma, LV Prasad Eye Institute, Kallam Anji Reddy Campus, Hyderabad, IND

**Keywords:** glaucoma progression, normal tension glaucoma, oct-angiography, vessel density, visual fields

## Abstract

Purpose: To investigate the clinical characteristics of normal-tension glaucoma (NTG) in patients under 40 years of age, aiming to identify distinct patterns and risk factors associated with progression in early-onset NTG.

Methods: A retrospective study was conducted on NTG patients under 40 years of age seen at a tertiary eye care center from 2006 to 2023. Details that were retrieved included visual field indices, intraocular pressure at baseline, central corneal thickness, refractive error, optical coherence tomography (OCT) & OCT angiography (OCT-A) parameters, ocular and systemic associations, and visual field progression on glaucoma progression analysis. Data were statistically analyzed using multivariate regression for predictors of progression.

Results: A total of 41 eyes from 26 NTG patients under 40 years (mean age = 32 ± 5.1 years; 22.5% female) were studied. Twelve had bilateral NTG, while 14 were unilateral (seven showing pre-perimetric changes and seven being normal or disc suspects). Affected eyes had significantly worse mean deviation (MD) (-10 ± 8.1 dB) and visual field index (VFI) (78 ± 25.9%) compared to unaffected eyes (-1 ± 1.1 dB and 92 ± 7.8%, p = 0.02). Low-to-moderate myopia was observed in 72%, while high myopia (>6 dioptres) was present in six eyes (three patients). Progression occurred in nine eyes (rate of progression = -3 ± 1.9 dB, including five eyes with high myopia), with high myopia and reduced macular VD being significant predictors (β = -0.6, p = 0.03; β = 0.3, p = 0.002).

Conclusion: NTG in individuals under 40 years of age is characterized by significant changes in macular vessel density. High myopia and reduced macular vessel density on OCT-A may be risk factors for the progression of NTG in young patients.

## Introduction

Normal-tension glaucoma (NTG) is a subtype of primary open-angle glaucoma characterized by progressive optic neuropathy and visual field loss despite intraocular pressure (IOP) measurements within the statistically normal range [1,2]. While NTG is more commonly diagnosed in older populations, its occurrence in younger individuals under 40 years of age is increasingly recognized. Early-onset NTG presents unique challenges due to its potential for rapid disease progression, the longer lifetime cumulative burden of vision loss, and differences in underlying mechanisms compared to NTG in older individuals [3,4].

Despite advancements in imaging and diagnostic technologies, the underlying mechanisms and distinguishing features of NTG in young patients remain insufficiently explored [1-6]. Factors such as vascular dysregulation, structural vulnerabilities in the optic nerve head, and microvascular impairments detectable by optical coherence tomography angiography (OCT-A) are thought to contribute to disease pathogenesis [2,3,5]. However, comprehensive studies focusing on the retinal nerve fiber layer (RNFL), macular ganglion cell complex (GCC), and optic nerve microvasculature in this age group are lacking. This study aims to analyze the clinical and imaging characteristics of NTG in individuals under 40 years of age and seeks to identify risk factors responsible for progression in NTG of the young.

## Materials and methods

This study was a retrospective analysis of patients diagnosed with NTG under the age of 40 at two tertiary eye care centers in East and South India from May 2006 to June 2023. This was approved by the institutional review board and followed the tenets of the Declaration of Helsinki with a waiver of consent for retrospective data analysis. Participants were recruited from glaucoma clinics and met the inclusion criteria of documented glaucomatous optic neuropathy (focal rim thinning or notch, with or without disc hemorrhages at any visit, RNFL defect) with corresponding visual field defects on standard automated perimetry, normal IOP below 21 mmHg without treatment, and an open anterior chamber angle on gonioscopy. Patients with secondary causes of glaucoma (traumatic optic neuropathy), disc suspects (without RNFL defects, localized or focal rim thinning, normal IOP, and visual field) with normal visual field, or systemic conditions affecting the optic nerve (neurodegenerative disorders, ischemic optic neuropathy, or compressive neuropathies) were excluded.

Details that were retrieved from the hospital database included best-corrected visual acuity (BCVA), IOP measurements with Goldmann applanation tonometry, slit-lamp biomicroscopy, fundus examination, and central corneal thickness measurements. Visual field testing was performed using the Humphrey Field Analyzer 3 (Zeiss, San Leandro, CA; 24-2 SITA FAST program), with mean deviation (MD), pattern standard deviation (PSD), and visual field index. The Topcon swept-source optical coherence tomography (OCT) system (DRI OCT Triton Plus, Topcon Corporation, Tokyo, Japan) was used to examine all selected eyes, performing a 7 mm 3D macular cube protocol, and the optic disc was evaluated using a 6 mm 3D disc protocol. Swept-source optical coherence tomography angiography (SS-OCTA) (OCT Topcon ImageNet 6, DRI OCT Triton, Topcon Corporation, Tokyo, Japan) imaging using the Angio 4.5 × 4.5 mm protocol captures both the macula and optic disc. OCT parameters that were retrieved and analyzed included RNFL thickness and macular ganglion cell complex-inner plexiform layer (GCC-IPL). Microvascular density in the peripapillary and macular regions on OCT-A was employed to evaluate vessel density (VD) using built-in processing software, with manual corrections made for motion artifacts. The vessel density is defined as the ratio of the area occupied by vessels with blood flow to the total measured area.

Statistical analysis included comparisons between progressors and non-progressors using Student’s t-test or Mann-Whitney U-test, depending on data distribution. Associations between RNFL thinning, GCC loss, vascular density, ocular associations, and visual field indices were analyzed using Pearson or Spearman correlation coefficients. Since each eye in NTG behaves differently with asymmetric damage being common, each eye was considered as a separate unit. Multivariable regression was conducted to identify independent predictors of disease severity with ganglion cell inner plexiform layer (GCIPL), RNFL, and MD evaluated in different models owing to collinearity. A p-value < 0.05 was considered statistically significant.

## Results

A total of 41 eyes of 26 participants were included in the study, comprising 12 bilateral NTG and 17 unilateral NTG under the age of 40 years (mean age: 33 ± 5.1 years; 23% female). Of 17 unilateral, seven eyes had pre-perimetric changes in the less affected eye, while the contralateral eye was disc suspect or normal in the rest. The MD or visual field index (VFI) was significantly lower in affected eyes in NTG (-10 ± 8.1 dB and 78 ± 25.9%, respectively) compared to eyes with pre-perimetric damage or the contralateral normal eyes of unilateral NTG (-1 ± 1.1 and 92 ± 7.8%, respectively, p = 0.02). The mean baseline IOP was 17 ± 2.6 mmHg, with observation without medications chosen for 17 of 41 eyes and treatment in 24 eyes (17 eyes on one medication, five eyes on two medications, and two eyes on three medications) at baseline (Table 1). The mean IOP did not differ significantly between the eyes on treatment and those on observation only (14 ± 3.1 mm Hg versus 12 ± 2.8 mm Hg, respectively, p = 0.8). The mean central corneal thickness (CCT) was 528 ± 23.9 µ, which was not statistically different between affected eyes (524 ± 21.6 µ) versus unaffected (520 ± 20.8 µ) eyes (p = 0.6).

**Table 1:** Baseline demographics of patients under 40 years with normal-tension glaucoma.

Variable	Mean ± standard deviation
Age at diagnosis (years)	33 ± 5.2
Male:female	20:6
Central corneal thickness (µ)	528 ± 23.9
Unilateral/bilateral eyes (n)	17:24
Baseline intraocular pressure (mm Hg)	17 ± 2.6
Mean deviation (dB)	-10 ± 8.1
Visual field index (%)	78 ± 25.9

Low-to-moderate myopia was found in 72% of the eyes (mean spherical equivalent of -2 ± 1.3 dioptres) while high myopia was found in six eyes of three patients (mean spherical equivalent of -6 ± 3.7 dioptres). Systemic associations, including diabetes and hypertension, were observed in seven patients with congenital hypertrophy of the retinal pigment epithelium, seen in one patient. A family history of glaucoma was not present in any of the patients in this cohort.

OCT was available in 14 of 26 patients, while OCT-A was available in five patients. Nine eyes progressed on guided progression analysis (GPA) over a mean period of 3 ± 2.2 years. Spectral-domain optical coherence tomography (SD-OCT) showed that NTG patients exhibited significant thinning of the RNFL in the superior and inferior quadrants (average RNFL thickness: 56 ± 12.5 µm, with reduced macular GCIPL thickness of 29 ± 8.04 µm than non-progressors) (Table 2).

**Table 2:** Comparison of clinical characteristics in patients <40 years with normal-tension glaucoma with and without glaucoma progression (see text for definition of glaucoma progression). * OCT was available in 14 of 26 patients, and OCT-A was available in five of 26 patients (see text for full description). ^ Mann-Whitney U test or unpaired Student's t-test RNFL: retinal nerve fiber layer; GCIPL: ganglion cell inner plexiform layer.

	Progressors (N = 9)	Non-progressors (N = 32)	P-value^
Age (years)	35 ± 4.6	32 ± 5.08	0.2
Mean deviation (dB)	-10 ± 8.7	-9 ± 5.9	0.
Visual field index (%)	76 ± 27.6	84 ± 18.4	0.8
Central corneal thickness (µ)	536 ± 22.7	526 ± 24.3	0.4
RNFL (µm)*	56 ± 12.5	77 ± 7.7	0.1
GCIPL (µm)	29 ± 8.04	64 ± 1.7	0.05
Rate of progression (dB/year)	-3 ± 1.9	0.02 ± 0.5	<0.001
Macular vessel density (%)	32 ± 2.2	40 ± 1.4	0.04
High myopia	5	1	0.002

OCT-A revealed a significant reduction in vessel density (VD) in five NTG patients, particularly in the peripapillary region (40 ± 2.4) and superficial macular layers (32 ± 2.2). Reduced macular VD correlated strongly with GCC thinning (r = 0.62, p < 0.01) and visual field mean deviation (r = 0.58, p < 0.01).

The rate of progression in nine eyes that progressed was -3 ± 1.9 dB, while that in non-progressors was -0.02 ± 0.5 dB. Five of the nine eyes (55.5%) that progressed were high myopes with a mean GCIPL and RNFL thickness of 53 ± 12.4 µm and 32 ± 18.2 µm, respectively. The CCT, baseline age, MD, VFI, and OCT parameters were not statistically different from non-progressors, while the progressors had a greater proportion of eyes with high myopia and a reduced macular vessel density (Table 2).

**Figure 1:**
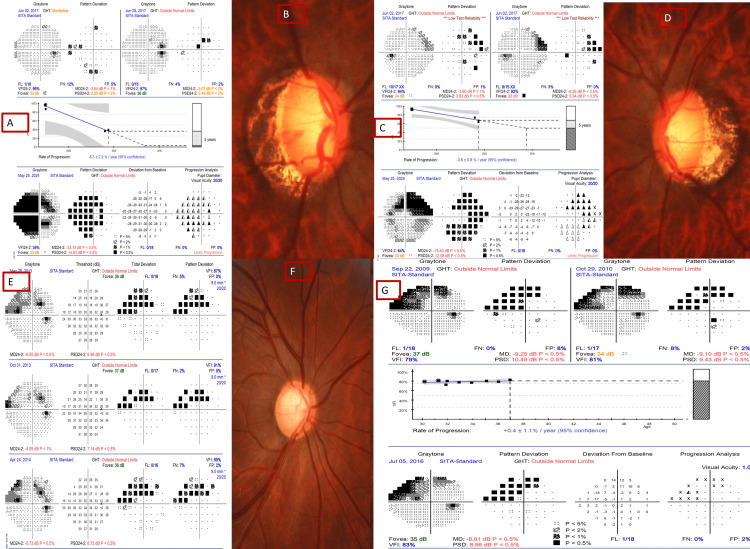
The fast rate of progression on guided progression analysis and myopic discs in the right (A, B) and left eyes (C, D) of a patient with high myopia (-11 dioptre spherical equivalent) seen over seven years versus the stable visual field/disc over a long period in non-progressors (E, F) with low myopia (spherical equivalent -1.5 D) and another patient with low myopia (G). VFI: visual field index; FP: false positive; FN: false negative; GHT: glaucoma hemifield test.

Figure 1 shows the rate of progression (-8dB to -3dB/year) and disc features in patients with NTG in progressors with high myopia versus non-progressors.

**Figure 2:**
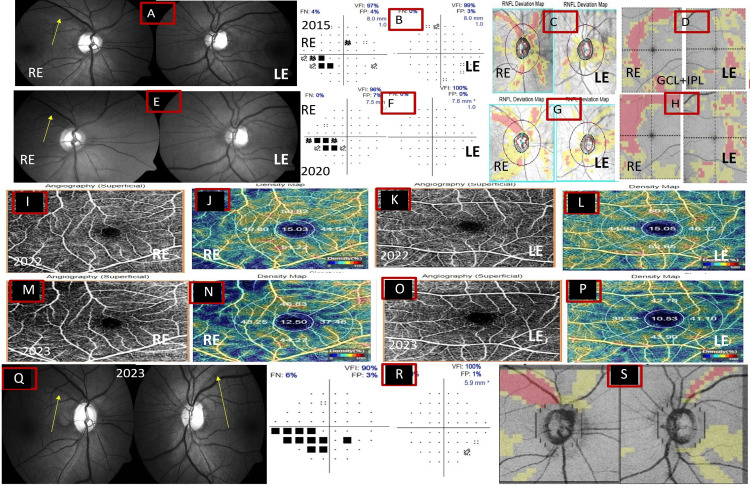
Long-term changes seen in the right and left eyes in a 31-year-old patient with NTG over a follow-up of five years (above panels represent images in 2015, below panels in 2020), analyzed using fundus, OCT, and OCT-angiography. Panel A shows significant disc damage with superotemporal RNFL defects (yellow arrows) in the right eye (A), while the left eye (E) has early damage. Panels B and F show the corresponding visual defects during the same period in both eyes (above panels of 2015, below panels of 2020). On OCT, the deviation map shows the RNFL defects in the supertemporal quadrant in the right eye that have increased in 2020 (C, G), while the left eye remains stable. The GCIPL thickness map of the right and left eye (D, H) during the same periods shows slow progressive thinning of the GCIPL thickness in both eyes. OCT angiography (I-P) shows the foveal avascular zone and macular vessel density in both eyes. The superficial macular vessel density (J and N; L and P) shows a decrease in the superior quadrant almost eight months before the visual field changes or the evident progression seen on fundus at seven years (Q), visual field (R), and OCT (S) compared with macular vessel density in 2022 and 2023 in both eyes (J and N for the right eye; L and P for the left eye). NTG: normal-tension glaucoma; OCT: optical coherence tomography; RNFL: retinal nerve fiber layer; GCIPL: ganglion cell inner plexiform layer; RE: right eye; LE: left eye; VFI: visual field index; FP: false positive; FN: false negative.

Figure 2 shows a patient with disc damage, early visual field defects, reduced RNFL thickness, and GCIPL thickness, which showed progression in both eyes over time. Interestingly, the macular VD and GCIPL showed changes before the increase in RNFL defect width and visual progression in both eyes, preceding the visual field by eight months, though the IOP remained stable at all visits and all other vascular associations were ruled out.

Multivariate regression identified macular vessel density reduction (β = -0.6, p = 0.03) and high myopia (β = 0.3, p = 0.002) as significant predictors of progression in this cohort (Table 3).

**Table 3:** Multivariate regression showing predictors of visual field progression in NTG patients under 40 years. Note: Mean deviation and the visual field index were run in different models owing to collinearity. NTG: normal-tension glaucoma; GCIPL: ganglion cell inner plexiform layer.

	Coefficient	P-value
Age (years)	-0.4	0.08
Mean deviation (dB)	-0.4	0.3
Visual field index (%)	0.2	0.06
GCIPL thickness (µm)	-0.8	0.06
Myopia > 6 dioptres	0.6	0.03
Vessel density reduction > 10%	0.3	0.002

## Discussion

The findings of this study suggest the role of GCIPL thinning and macular VD reduction in the pathophysiology and progression of NTG. While these changes precede the visual field changes by two to three months, it is uncertain if VD changes are a cause or effect of disease progression.

Reduced macular VD, as quantified by OCT-A, points to a microvascular component in NTG pathogenesis. The close association between VD and GCIPL thickness suggests that ischemic or vascular insufficiency may contribute to neuronal degeneration. These findings align with emerging evidence that vascular dysregulation and microvascular impairments play a central role in NTG, especially in younger populations, where traditional risk factors like elevated intraocular pressure are often absent [2,5-11]. The combined assessment of GCIPL thickness and macular VD may therefore serve as a more robust means of monitoring NTG in young patients [11,12].

High myopia and reduced macular VD emerged as significant factors influencing the progression of NTG in young patients, highlighting the interplay between structural and vascular factors in disease pathogenesis [1-3,11,12]. Though GCIPL thickness correlated with macular VD, GCIPL thickness was not significantly different between progressors and non-progressors. High myopia and associated structural changes induced by stretching of the ocular coats increase the susceptibility of the optic nerve head to mechanical stress and deformation [9,12-15]. While this in itself may predispose to NTG in younger patients with myopia, the correlation between myopia and macular VD reduction suggests that myopic eyes may have compromised microvascular circulation due to stretching and thinning of retinal layers, particularly in the macular region. The findings of this study suggest the adjunctive use of OCT and OCT-A to discern early changes in NTG patients with early onset. These vascular changes may be the markers for vascular dysfunction even before structural changes in RNFL/GCIPL and visual field, though this needs longitudinal studies to confirm. The combination of structural vulnerability from myopia and microvascular insufficiency likely exacerbates retinal ganglion cell damage, accelerating disease progression in young NTG patients.

We did not evaluate OCT-A in all eyes, which was available only a few years back, which precludes generalization of the results to all patients. Also, we did not evaluate axial length in all eyes since that is a marker for eye stretching rather than a myopic refractive error. Systemic associations are known in NTG eyes, though these have distinct changes in OCT or OCT-A, and we do not believe these could confound the results in this study, as was confirmed with the absence of these factors associated with progression on multivariate analysis in this study. It may be possible that a few stable eyes with moderate myopia may also progress over a longer follow-up, which needs longitudinal studies to evaluate long-term changes. We also did not compare this cohort with NTG in >40 years old patients in this retrospective study, since the pathogenesis in the latter is known and well established. Prospective studies comparing the changes in these with age-compared changes in RNFL or macular parameters may confirm the vascular dysregulation in NTG in young patients.

## Conclusions

Nevertheless, reduced macular VD and high myopia may be predictors for progression in NTG patients who present before 40 years. Prospective studies comparing the changes in these with age-compared changes in RNFL or macular parameters may confirm the vascular dysregulation in NTG in young patients. Adjunctive use of OCT and OCT-A in NTG eyes, especially in eyes with early-onset glaucoma, may help identify changes before functional abnormalities in the visual field.
